# Prenatal Diagnosis of Chromosomal Mosaicism in Over 18,000 Pregnancies: A Five-Year Single-Tertiary-Center Retrospective Analysis

**DOI:** 10.3389/fgene.2022.876887

**Published:** 2022-05-16

**Authors:** Shuyuan Li, Yiru Shi, Xu Han, Yiyao Chen, Yinghua Shen, Wenjing Hu, Xinrong Zhao, Yanlin Wang

**Affiliations:** ^1^ The International Peace Maternity and Child Health Hospital, School of Medicine, Shanghai Jiao Tong University, Shanghai, China; ^2^ Shanghai Key Laboratory of Embryo Original Diseases, Shanghai, China

**Keywords:** chromosomal mosaicism, prenatal diagnosis, ultrasound malformations, inconsistent results, termination of pregnancy

## Abstract

**Background:** Chromosomal mosaicism (CM) is a common biological phenomenon observed in humans. It is one of the main challenges in prenatal diagnosis due to uncertain outcomes, especially when fetal ultrasonographic features appear normal. This study aimed to assess the phenotypic features of CM detected during prenatal diagnosis and the risk factors affecting parents’ pregnancy decisions.

**Materials and methods:** A retrospective cohort study involving 18,374 consecutive pregnancies that underwent prenatal diagnosis by karyotyping, fluorescence *in situ* hybridization (FISH), or chromosome microarray analysis (CMA) was conducted. The association of risk factors with malformations detected by ultrasound and pregnancy outcomes was assessed using the chi-square test and binary logistic regression. Discordant results between the different methods were identified and further analyzed.

**Results:** During this five-year period, 118 (0.6%) patients were diagnosed with CM. The incidences of CM in the chorionic villus, amniotic fluid, and umbilical cord blood were 3.2, 0.5, and 0.7%, respectively. The frequency of ultrasound malformations in individuals with a high fraction of autosomal CM was significantly higher than that in other groups (62.5% vs. 21.4–33.3%, all *p* <0.05). Inconsistent results between karyotyping and CMA/FISH were observed in 23 cases (19.5%). The risk of pregnancy termination in cases with ultrasound malformations, consistent results, autosomal CM, or a high CM fraction increased with an odds ratio of 3.09, 8.35, 2.30, and 7.62 (all *p* <0.05). Multiple regression analysis revealed that all four factors were independent risk factors for the termination of pregnancy.

**Conclusion:** Patients with a high fraction of autosomal CM are more likely to have ultrasound malformations. Inconsistent results between different methods in CM are not rare. Ultrasound malformations, consistent results between different methods, autosomal CM, and a high CM fraction were independent risk factors for the choice to terminate pregnancies.

## Introduction

Chromosomal mosaicism (CM) refers to the presence of two or more chromosomally different cell lines in an individual derived from a single zygote (Eggermann et al., 2015). It is a biological phenomenon in humans that may occur through a variety of mechanisms, including chromosome non-disjunction, anaphase lagging, trisomy rescue, and endoreplication (Taylor et al., 2014). It has been reported that CM occurs frequently during human pre-implantation development, with a prevalence of 15–75% in cleavage-stage embryos and 3–34% in blastocysts (Harton et al., 2017). However, with embryo development, CM is assumed to be less pervasive (Popovic et al., 2020). Previous studies demonstrated that CM was found in approximately 1–4% of prenatal diagnoses performed by chorionic villus sampling (CVS) and in approximately 0.1–0.3% of amniocentesis (Hsu et al., 1996; Carey et al., 2014; Grati et al., 2017; Gu et al., 2018; and Lund et al., 2020). Nevertheless, CM is still one of the main challenges in prenatal diagnosis due to uncertain outcomes, especially when fetal ultrasonographic features appear normal (Wallerstein et al., 2015).

In recent years, karyotyping, fluorescence *in situ* hybridization (FISH), and chromosome microarray (CMA) analysis have been widely used in the prenatal diagnosis for chromosomal analysis. Karyotyping, which requires cell culture, is a conventional cytogenetic test with a resolution of 5–10 Mb. Unlike karyotyping, FISH and CMA are performed in uncultured cells or DNA extracted from uncultured cells, respectively. Generally, FISH is used to detect numerical aberrations of chromosomes 13, 18, 21, X, and Y rapidly, whereas CMA can detect aneuploidy, microduplications, and microdeletions throughout the genome. Unlike karyotyping and FISH, which require manual counting of the chromosome composition, the result of CMA can be achieved automatically through a bioinformatic analysis. All the aforementioned differences in these methods can lead to inconsistencies in the results, especially in the case of CM. The discordant results further aggravate the challenges of genetic counseling for CM in prenatal diagnosis.

This study aimed to assess the incidence and characteristics of CM detected by karyotyping, FISH, and/or CMA in more than 18,000 consecutive pregnancies referred to our center for prenatal diagnosis over a five-year period, with a focus on their phenotypic features and the risk factors affecting the parents’ pregnancy decisions, and further comparing the discordant results identified by different methods.

## Materials and Methods

### Patients

**FIGURE 1:**
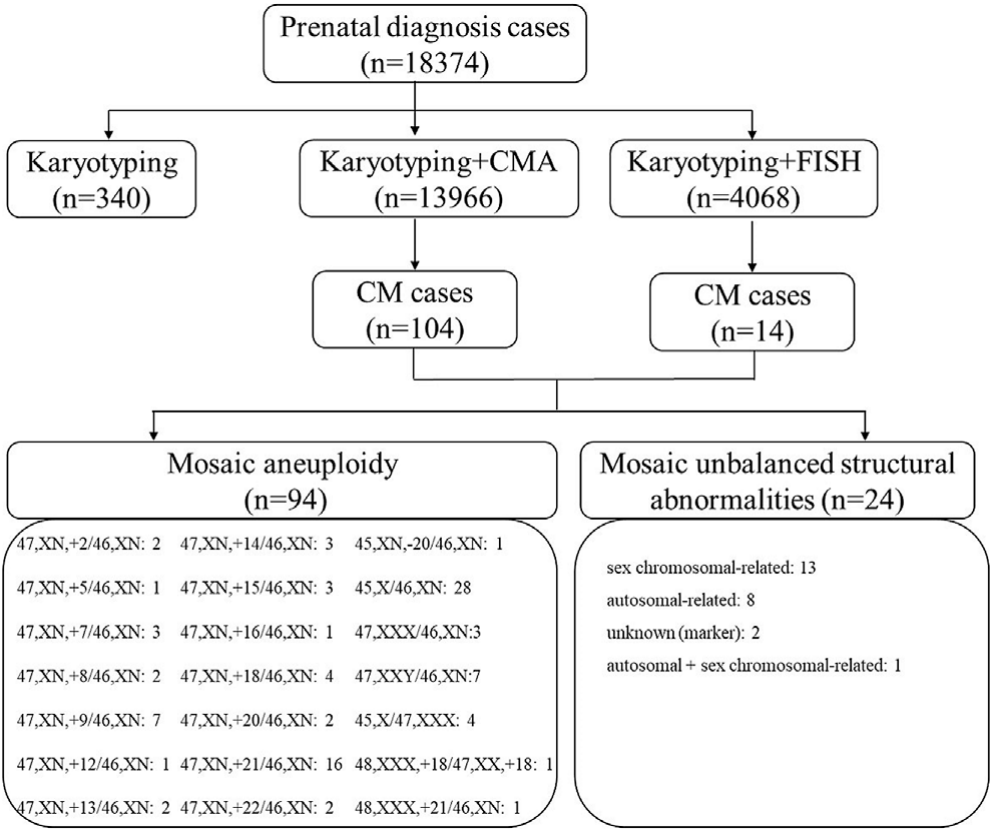
Flow chart of fetuses with CM at our center in a five-year period between January 2016 and December 2020. CM, chromosomal mosaicism; CMA, chromosomal microarray; FISH, fluorescence *in situ* hybridization; T, trisomy.

This retrospective single cohort study was conducted in the reproductive genetic center of the International Peace Maternal and Child Health Hospital (IPMCH) of Shanghai Jiao Tong University School of Medicine. From January 2016 to December 2020, 18,374 fetuses were consecutively referred to our center for invasive prenatal diagnosis. According to the gestational age, fetal samples were obtained using CVS (*n* = 823), amniocentesis (*n* = 16,419), or umbilical cord blood (UCB) sampling (*n* = 1,132). All fetal samples were analyzed by karyotyping (*n* = 340, 1.8%), karyotyping and CMA (*n* = 13,966, 76.0%), or karyotyping and FISH (*n* = 4,068, 22.1%). Among them, 118 cases diagnosed with CM using at least one method were selected and further analyzed in this study, including 113 singleton pregnancies and five twin pregnancies. In twin pregnancies, only one fetus was affected in each pair. Among the 118 cases, 104 were diagnosed by karyotyping and CMA, whereas 14 were diagnosed by karyotyping and FISH (Figure 1). At our center, all cases diagnosed with CM were recommended for a more detailed ultrasonographic examination to further identify structural abnormalities in the fetuses. All cases were further consulted regarding prognosis and were additionally followed up for clinical outcomes.

### Karyotyping Analysis, Fluorescence *In Situ* Hybridization, and Chromosomal Microarray

Cells were cultured and prepared for conventional G-banding karyotyping (550-band resolution), according to the standard protocol for all 18,374 fetal samples. Generally, at least 15 metaphase cells were assessed for numerical abnormalities of chromosomes, and five metaphase cells were carefully examined by experienced technicians to detect structural chromosomal abnormalities. If abnormal karyotypes were identified within the 15 metaphase cells, additional images were captured, and a total of 50 metaphases were karyotyped for each sample if possible. CM was diagnosed when ≥2 cells with the same abnormality were observed in two independent culture vessels.

FISH was performed in uncultured cells using commercially available probes for chromosomes 13, 18, 21, X, and Y, according to the manufacturer’s instructions (Beijing GP Medical Technologies, Beijing, China). At least 50 interphase nuclei per target probe were evaluated for each sample. If 90% of the detected cells were normal, the sample was classified as normal. If 60% of the cells were abnormal, the sample was considered abnormal. In cases in which there was any doubt, the number of cells evaluated increased to 100. Mosaic was suspected when 10–60% of the cells were aberrant, and the results were reported as uninformative through FISH.

Genomic DNA was isolated according to standard procedures (Li et al., 2019). Quantitative fluorescent (QF)-PCR (R1004T; GENESKY, Shanghai, China) was used for a potential maternity contamination analysis in suspicious samples. Approximately all CMA analyses were performed on direct (uncultured) specimens, except those with maternal cell contamination. CMA was performed using Affymetrix CytoScan 750 K Array (Affymetrix, Inc., Santa Clara, CA, United States). Results were analyzed by Affymetrix Chromosome Analysis Suite software (ChAS) version 3.1. Genomic coordinates were based upon the UCSC human Genome Browser release of February 2009 (GRCh37/hg19). CNVs of a region of at least 100 kb with a minimum of 50 markers were analyzed carefully. The interpretations of CNVs were performed according to the guidelines (Kearney et al., 2011; Riggs et al., 2019). The level of mosaicism was obtained from the median Log2Ratio value calculated by the piece of software, and CM was reported when the level was between 20 and 70%.

### Statistical Analysis

Continuous variables are presented as the mean ± standard deviation or median (range). Categorical variables are summarized as numbers (percentage). The association of risk factors with ultrasound malformations and pregnancy outcomes was assessed using the chi-square test and binary logistic regression. Increased nuchal translucency (NT), echogenic intracardiac focus, choroid plexus cysts, echogenic bowel, mild ventriculomegaly, thickened nuchal fold, mild pyelectasis, single umbilical artery, hypoplastic nasal bone, and enlarged cisterna magna were defined as ultrasound soft markers (Li et al., 2020). Both ultrasound structural anomalies and ultrasound soft markers were defined as ultrasound malformations. All CMs related to autosomal chromosomes were classified into the group of autosomal abnormalities, whereas CMs related to sex chromosomes were classified into the group of sex chromosomal abnormalities. With respect to the CM fraction, we defined a fraction greater than or equal to 50% as high and others as low (Bellil et al., 2020; Capalbo et al., 2021). As the results of karyotyping which were counted manually may introduce human error, the classification of the CM fraction was performed based on the results of CMA. In cases with normal CMA but an abnormal karyotype, the fraction of karyotyping results was used. For cases analyzed by karyotyping and FISH, the fraction of the FISH results was used. Inconsistent results between karyotyping and CMA/FISH were also analyzed. All analyses were performed by SPSS statistical software ver. 22.0 (IBM, Armonk, NY, United States). A *p* value <0.05 (two sides) was statistically significant.

## Results

### Clinical Characteristics

The clinical characteristics of 118 patients with CM are presented in Table 1. The incidences of CM in CVS, amniotic fluid (AF), and UCB were 3.2% (26/823), 0.5% (84/16,419), and 0.7% (8/1,132), respectively. Among the 26 CVS cases, five were subjected to amniocentesis, and the AF results were normal, indicating confined placental mosaicism (CPM). Ultrasound malformations were observed in 44 fetuses (37.2%). Among the 14 cases with ultrasound soft markers, increased NT (*n* = 12) was the most common type.

**TABLE 1:** Clinical characteristics of 118 patients with CM in this study.

	*n* (%)/median (range)
N	118
Maternal age, years	32 (18, 45)
Gestational age, weeks	18 (12, 30)
Invasive procedures	
CVS	26 (22.0)
AF	84 (71.2)
UCB	8 (6.8)
Indications	
Positive non-invasive prenatal test	32 (27.1)
Ultrasound structural anomalies	30 (25.4)
Advanced maternal age (≥35)	20 (16.9)
Ultrasound soft markers	14 (11.9)
Abnormal biochemical screening	9 (7.6)
Adverse history of pregnancy	6 (5.1)
Others	7 (5.9)
Type of CM	
Mosaic aneuploidy	94 (79.7)
Autosomal trisomy * ^a^	49 (41.5)
Sex chromosomal monosomy ^b^	28 (23.7)
Sex chromosomal trisomy ^b^	10 (8.5)
Sex chromosomal monosomy + trisomy ^b^	4 (3.4)
Autosomal trisomy + sex chromosomal trisomy ^a^	2 (1.7)
Autosomal monosomy ^a^	1 (0.8)
Mosaic unbalanced structural abnormalities	24 (18.6)
Sex chromosomal–related ^b^	13 (11.0)
Autosomal-related ^a^	8 (6.8)
Unknown (marker)	2 (1.7)
Autosomal + sex chromosomal–related ^a^	1 (0.8)

*Include one fetus with mosaic trisomy 15 and 22q11.2 deletion.

a classified into the group of autosomal abnormalities.

b classified into the group of sex chromosomal abnormalities.

CM, chromosomal mosaicism; CVS, chorionic villus sampling; AF, amniotic fluid; UCB, umbilical cord blood.

### Types of Chromosomal Mosaicism and Their Associations With Ultrasound Malformations

Overall, 94 (79.7%) mosaic aneuploidy cases and 24 (20.3%) mosaic unbalanced structural abnormalities were identified (Table 1 and Figure 1). Of the 94 mosaic chromosomal aneuploidy cases, mosaic autosomal trisomy was the most common type (*n* = 49). Among the 49 cases of autosomal trisomy CM, the most common type was trisomy 21 (*n* = 16), followed by trisomy 9 (*n* = 7) and trisomy 18 (*n* = 4) (Figure 1). With respect to mosaic unbalanced structural abnormalities, 13 were sex chromosomal–related abnormalities, while eight were autosomal–related abnormalities. Notably, two fetuses with mosaic marker chromosomes but an unknown source and one fetus with partial chromosome 8 duplication and Y chromosome deletion were identified.

To explore the effects of CM types and CM fractions on fetal phenotypes, we further compared the frequencies of ultrasound malformations among fetuses with different types and fractions of CM. As shown in Table 2, the frequency of ultrasound malformations in cases with mosaic autosomal abnormalities was higher than that in cases with sex chromosomal abnormalities, although the difference was not statistically significant, partially because of the small sample size (44.3 vs. 27.3%; *p* = 0.06). The same result was observed when comparing cases with a high fraction of CM and those with a low fraction of CM (45.1 vs. 28.4%; *p* = 0.06).

**TABLE 2:** Associations of CM types and CM fractions with ultrasound malformations.

	Cases with ultrasound malformations, *n* (%)	Cases without ultrasound malformations, *n* (%)	*p*
CM types			
Autosomal	27 (44.3)	34 (55.7)	0.06
Sex chromosomal	15 (27.3)	40 (72.7)	
CM fraction			
High (≥50%)	23 (45.1)	28 (54.9)	0.06
Low (<50%)	19 (28.4)	48 (71.6)	
CM types + CM fraction			0.006^a^
Autosomal high	15 (62.5)	9 (37.5)	
Autosomal low	12 (32.4)	25 (67.6)	0.02^b^
Sex chromosomal high	9 (33.3)	18 (66.7)	0.04^b^
Sex chromosomal low	6 (21.4)	22 (78.6)	0.003^b^

a *p* for trend.

b Compared to the group of autosomal high.

CM, chromosomal mosaicism.

We further compared the frequency of ultrasound malformations among groups with a high fraction of autosomal CM, a low fraction of autosomal CM, a high fraction of sex chromosomal CM, and a low fraction of sex chromosomal CM. The frequency of ultrasound malformations in individuals with a high fraction of autosomal CM was significantly higher than that in the other groups (62.5% vs. 21.4–33.3%, all *p* <0.05). A significant trend was observed for the frequency of ultrasound malformations among the four groups (*p* for trend = 0.006).

### Inconsistent Results Between Karyotyping and Chromosomal Microarray /Fluorescence *In Situ* Hybridization

Inconsistent results between karyotyping and CMA/FISH were identified in 23 cases (19.5%), including seven cases (26.9%) derived from CVS and 16 cases (19.0%) derived from AF (Table 3).

**TABLE 3:** Inconsistent results in fetuses with CM between the karyotype and CMA/FISH.

Case ID	Sample	UM	1st results		2nd results			Pregnancy outcome
			Karyotype	CMA	Sample	Karyotype	CMA and/or FISH	
1	CVS	No	46, XN	arr (X) × 1–2	AF	46, XN	arr (1–22) × 2, (X, N) × 1	Full-term delivery
2	CVS	Increased NT (3.5 mm)	46, XN	arr (X) × 1, (Y) × 0–1	AF	46, XN	arr (1–22) × 2, (X, N) × 1	Full-term delivery
3	CVS	Increased NT (3.1 mm)	46, XN	arr (7) × 2–3	AF	46, XN	arr (1–22) × 2, (X, N) × 1	Full-term delivery
4	CVS	Hydrops	46, XN	arr (X) × 1, (Y) × 0–1	AF	46, XN	arr (1–22) × 2, (X, N) × 1	TOP
5	CVS	anencephaly	46, XN	arr (7) × 2–3				TOP
6	CVS	No	45, X (9)/46, XX (41)	arr (1–22) × 2, (X, N)×1				Full-term delivery
7	CVS	Hydrops	46, XN, der (10) (pter→q26::?)(7)/46, XN (46)	arr (1–22) × 2, (X, N) × 1				TOP
8	AF	No	46, XN	arr (X) × 1–2	AF/PB	46, XN	arr (1–22) × 2, (X, N) × 1	Full-term delivery
9	AF	No	46, XN	arr (15) × 2–3	AF	46, XN	arr (15) × 2–3 nuc ish (CSP15 × 3) (21/100)^a^	Preterm delivery (33 weeks)
					UCB	46, XN	arr (1–22) × 2, (X, N) × 1	
							Normal^a^	
					Urine		seq (15) × 2–3^b^	
					OMC		seq (15) × 2–3^b^	
					UCR		seq (15) × 2–3^b^	
					UC		Normal^b^	
					PB		Normal^b^	
10	AF	MM	46, XN	arr (14) × 2–3				TOP
11	AF	CHD	46, XN	arr (16) × 2–3				TOP
12	AF	echogenic bowel	46, XN	arr (7) × 2–3	UCB	46, XN		Lost to follow-up
13	AF	Increased NT (3.0 mm)	45, X (4)/46, XN (26)	arr (1–22) × 2, (X, N) × 1				Full-term delivery
14	AF	No	47, XN.+20 (9)/46, XN (57)	arr (1–22) × 2, (X, N) × 1				Full-term delivery
15	AF	No	47, XN,+20 (5)/46, XN (45)	arr (1–22) × 2, (X, N) × 1				Full-term delivery
16	AF	No	45, XN,-20 (3)/46, XN (47)	arr (1–22) × 2, (X, N) × 1				Full-term delivery
17	AF	No	47, XN,+?8 (3)/46, XN (15)	arr (1–22) × 2, (X, N) × 1				Preterm delivery (33 weeks)
18	AF	CCAM	45, X (3)/46, XN (37)	arr (1–22) × 2, (X, N) × 1				Full-term delivery
19	AF	MM	45, X (3)/46, XX (32)	arr (1–22) × 2, (X, N) × 1				TOP
20	AF	MM	47, XN,+5 (4)/46, XN (46)	arr (1–22) × 2, (X, N) × 1				TOP
21	AF	Increased NT (3.3 mm)	47, XN,+mar (5)/46, XN (45)	arr (1–22) × 2, (X, N) × 1				Full-term delivery
22	AF	VSD	47, XN,+mar (4)/46, XN (55)	arr (1–22) × 2, (X, N) × 1				Full-term delivery
23	AF	No	47, XX + 21 (3)/46, XX, (7)	Normal^a^	placenta	46, XN		Full-term delivery

a Analyzed by FISH.

b Copy number variation sequencing was performed in these samples obtained after birth.

CM, chromosomal mosaicism; CMA, chromosomal microarray; FISH, fluorescence *in situ* hybridization; UM, ultrasound malformations; CVS, chorionic villus sampling; AF, amniotic fluid; PB, peripheral blood; UCB, umbilical cord blood; TOP, termination of pregnancy; OMC, oral mucosal cells; UCR, umbilical cord root; UC, umbilical cord; CCAM, congenital cystic adenomatoid malformation; VSD, ventricular septal defect; MM, multiple malformations; CHD, congenital heart disease.

Among the seven CVS cases with inconsistent results, four had underwent amniocentesis, and the results were normal (cases 1–4), indicating CPM. Of the 16 AF cases, a subsequent genetic analysis was performed in only four cases (8, 9, 12, and 23). In case 8, a second amniocentesis was performed, and both karyotyping and CMA were normal. AF and UCB were redrawn in case 9; the results of CMA and FISH in uncultured AF were still mosaic trisomy 15, whereas the results of karyotyping, CMA, and FISH in UCB were normal. The fetus was delivered prematurely at 33 weeks because of maternal antepartum hemorrhage. After birth, copy number variation sequencing of peripheral blood, urine, oral mucosal cells, umbilical cord root, and umbilical cord (approximately 3 cm away from the root of the umbilical cord root) was performed. As shown in Table 3, a low fraction of mosaic trisomy 15 was detected in the urine, oral mucosal cells, and umbilical cord root, whereas no abnormalities were detected in the umbilical cord and peripheral blood. UCB puncture was performed in case 12, and the karyotype was normal. The karyotyping result of the placenta in case 23 was normal after birth. Cases 13–22 were AF cases where karyotyping was abnormal, but the CMA was normal. The discordant results may be due to the low mosaic fraction (<20%) for which the CMA cannot detect (cases 13–20) or may indicate that the marker chromosome was heterochromatin (cases 21–22). No further genetic testing was performed in these patients.

Fifteen of them were born. Except for a short stature below the 10th percentile of case 1 (at 2 years of age), the development of all other cases has been normal until the time of publication (6 months–4 years of age).

### Risk Factors Affecting the Parent’s Pregnancy Decision

Pregnancy outcomes were available for 115 cases [115 of 118 (97.5%)]. Overall, 81 (77.1%) cases had a termination of pregnancy (TOP). The effects of ultrasound malformations, inconsistent results of different methods, different types of CM, and CM fractions on the choice of TOP were examined. As shown in Table 4, the rate of TOP in cases with ultrasound malformations was significantly higher than that in cases without ultrasound malformations (83.7 vs. 62.5%, *p* = 0.02). Similar results were observed in cases with consistent results, autosomal CM, or a high CM fraction in comparison to those with inconsistent results, sex chromosomal CM, or a low CM fraction (79.6 vs. 31.8%, *p* = 1 × 10^−5^; 79.3 vs. 63.0%, *p* = 0.049; and 90.2 vs. 54.7%, *p* = 3 × 10^−5^). Compared to cases without ultrasound malformations, or with inconsistent results, sex chromosomal CM or a low fraction of CM, the risk of the TOP in those with ultrasound malformations, consistent results, autosomal CM, or a high CM fraction was increased with an odds ratio of 3.09 (95% CI: 1.21–7.90, *p* = 0.02), 8.35 (95% CI: 1.15–7.52, *p* = 5 × 10^−5^), 2.30 (95% CI: 1–5.34, *p* = 0.049), and 7.62 (95% CI: 2.68–21.70, *p* = 1 × 10^−4^), respectively. To further clarify the independent effects of these factors on pregnancy outcomes, multiple logistic regressions were conducted. The results showed that all four factors were independent risk factors for the TOP (Table 4).

**TABLE 4:** Effect of ultrasound features, CM types, CM fractions, and consistent or inconsistent results on pregnancy outcomes.

	N	TOP, *n* (%)	*p*	Or (95% CI)	*p*	Adj-OR*	Adj-p*
Ultrasound malformations							
Yes	43	36 (83.7)	0.02	3.09 (1.21, 7.90)	0.02	6.02 (1.48, 24.53)	0.01
No	72	45 (62.5)					
Consistent results							
Consistent	93	74 (79.6)	1 × 10^−5^	8.35 (2.98, 23.36)	5 × 10^−5^	14.0 (3.08, 63.59)	0.001
Inconsistent	22	7 (31.8)					
CM types							
Autosomal	59	47 (79.3)	0.049	2.30 (1, 5.34)	0.049	4.41 (1.41, 13.76)	0.01
Sex chromosomal	54	34 (63.0)					
CM fractions							
High (≥50%)	51	46 (90.2)	3 × 10^−5^	7.62 (2.68, 21.70)	1 × 10^−4^	6.18 (1.86, 20.58)	0.003
Low (<50%)	64	35 (54.7)					

*Covariates listed in the table were mutually adjusted.

CM, chromosomal mosaicism; TOP, termination of pregnancy; OR, odds ratio; CI, confidence interval.

## Discussion

In this study, we retrospectively analyzed 118 cases of CM detected at our center in a five-year period between January 2016 and December 2020 and found that approximately 62.8% of fetuses with CM had no abnormalities detected on ultrasound, further confirming that genetic counseling for CM is indeed a great challenge in the clinical setting (Zhang et al., 2021). To provide valuable information for genetic counseling and management of prenatal mosaic cases for clinicians and patients, we focused on the factors related to ultrasound malformations and pregnancy outcomes.

Our study demonstrated that compared to cases with autosomal CM, those with sex chromosomal CM were more likely to exhibit normal ultrasound finding and lead to a normal birth. In our study, 20 cases of sex chromosomal CM were born, including 10 cases of 45, X/46, XX; four cases of 45, X/46, XY; three cases of 47, XXY/46, XY; two cases of 47, XXX/46, XX; and one case of mosaic X chromosomal unbalanced structural abnormality. Except for one case that exhibited a short stature, the development of all other cases (6 months–3 years of age) was normal. Long-term follow-up and management of these cases is warranted as some of them may encounter abnormal sexual development (Dendrinos et al., 2015; Wong et al., 2021), autism spectrum disorder, or cognitive problems later in life (Vorsanova et al., 2021). Recently, Tuke et al. demonstrated that mosaic monosomy X showed reduced penetrance, and the management of women with 45, X/46, XX should be minimal in an adult population study (Tuke et al., 2019). Studies have also shown that compared to non-mosaic 47, XXY, the phenotypic symptoms in cases of XXY/XY mosaicism may present more mildly, and many cases fail to be identified (Samplaski et al., 2014; Davis et al., 2016). More population-based studies are needed to demonstrate the prevalence of abnormal phenotypes in individuals with sex CM to better guide clinical management and genetic counseling.

Unlike sex CM, there is no strong correlation between karyotypic and phenotypic abnormalities in autosomal CM (Wallerstein et al., 2015). We found that the frequency of ultrasound malformations in cases with a high fraction of autosomal CM was higher (62.5%) than that in cases with a low fraction of autosomal CM (32.5%; *p* = 0.02), indicating that a high mosaic fraction was a risk factor for adverse outcomes. Trisomy 9 mosaicism (T9M) is a rare chromosomal abnormality with a significant clinical variability (Pejcic et al., 2018). To date, no more than 200 cases of T9M have been reported (Li et al., 2021); surprisingly, seven cases with T9M were detected in 11,834 cases (0.06%) at our center, indicating that it was not rare in fetuses. Six of the seven cases chose TOP, whereas one fetus was born without any clinical defects, and no evident abnormalities were identified to date (3.5 years old). As the long-term growth trends of T9M vary widely, follow-up and management of the case is warranted. It is also worth mentioning that although the risk of abnormal outcomes of 47,+20/46 was very high (>60%) in a review conducted by Wallerstein et al. (2015), others reported that approximately 90–93% of cases with mosaic trisomy 20 (T20M) at the prenatal diagnosis have been associated with a normal phenotype (Hsu et al., 1987; Chen et al., 2020). In our study, two T20M cases were detected in AF by karyotyping, whereas the results of CMA were normal. Both patients were born normally, suggesting that the prognosis of T20M, which CMA cannot detect, was good, consistent with previous studies (Hsu et al., 1987; Chen et al., 2020).

Inconsistent results between different methods in CM were not rare and were observed in 19.5% of the cases in our study. The selective growth of the cells during culture and the detection ability of different methods for the low fraction of CM may be responsible for these inconsistent results (Cheung et al., 2007). Although we recommended the use of multiple methods, such as a combination of karyotyping, CMA, and FISH to further confirm the results before any irreversible decision was made, it was very difficult to implement, hindered by the uncertainty of clinical outcomes even after the second results were normal, as well as the high cost of genetic testing. Four of the seven CVS cases with inconsistent results underwent amniocentesis, and the results were normal, indicating CPM. In our case series, the incidence of CM in CVS was 3.2%, similar to the results reported by others using CMA (Gu et al., 2018; Lund et al., 2020). However, as most of these cases exhibited increased NT and/or fetal structural anomalies (23/26, 88.4%), the detected CM can partially explain the abnormal phenotypes (i.e., increased NT); amniocentesis was not performed in most of them. The ratio of CPM in our study could not be achieved. Recently, associations of CPM with negative developmental outcomes, including fetal growth restriction (71.7%), preterm birth (31.0%), and structural fetal anomalies (24.2%), have been reported, especially when chromosomes 2, 3, 7, 13, 15, 16, and 22 are involved (Eggenhuizen et al., 2021). In this circumstance, the CM detected in the CVS needed to be counseled with caution, even if it was found to be CPM. Unlike CVS, AF is considered to be the optimal specimen for fetal confirmation as it includes cells primarily from fetal anatomical districts, including the urogenital tract, respiratory tract, and epithelial systems, representing different embryological layers (Cremer et al., 1981). Partly because of the application of a high-resolution CMA, the incidence of CM in AF was 0.5% in our case series, which was slightly higher than previous reports (0.1–0.3%) (Hsu et al., 1996; Carey et al., 2014). It is generally accepted that UCB puncture is not required when CM is identified in AF as UCB cells are primarily derived from the mesoderm and can only reveal the mosaicism state of the mesoderm (Wieczorek et al., 2003). The negative UCB result could not negate the CM results of AF. However, in our study, two patients (case 9 with mosaic trisomy 15 and case 12 with mosaic trisomy 7) required UCB puncture due to anxiety. The UCB results in these two cases were normal. Although the outcome of case 12 was unknown, the development of case 9 was normal, indicating that a negative UCB result may be more prone to a good prognosis. More case evidence is required to demonstrate the significance of the UCB puncture in the case of CM identified in AF.

In our hospital, counseling on CM is provided by a geneticist at the prenatal diagnosis center. We observed that 77.1% of the patients decided to proceed with the TOP. Cases with ultrasound malformations, consistent results between karyotyping and CMA/FISH, autosomal CM, or a high fraction of CM were more likely to result in a TOP, whereas those with normal ultrasound, inconsistent results, sex chromosomal CM, or a low fraction of CM were likely to continue with the pregnancy and lead to normal birth. However, as CM is associated with many other abnormalities, including neuropsychiatric disorders, long-term monitoring and follow-up of these carriers are necessary.

It is worth mentioning that the sensitivity and specificity of ultrasound in the detection of malformations were important factors in our study. However, autopsy was not performed in the cases of TOP; therefore, we could not determine whether there were other structural abnormalities that were not detected by the prenatal ultrasound. However, for almost all live-birth cases, the results were consistent with the prenatal ultrasound findings, suggesting that the results of the prenatal ultrasound in our patients were reliable. In addition, although it does not affect our conclusions, it should be mentioned that mosaic unbalanced structural abnormalities with a fragment size smaller than 5 Mb would be missed in 14 cases of CM diagnosed by karyotyping and FISH.

In conclusion, our study demonstrated that patients with a high fraction of autosomal CM were more likely to have ultrasound malformations. Inconsistent results between different methods in CM are not rare. All four factors, including ultrasound malformations, consistent results between different methods, autosomal CM, and a high CM fraction, were independent risk factors for the choice of TOP. Further studies are warranted to provide more information for genetic counseling during prenatal diagnosis.

## Data Availability

The data presented in the study can be found in the ClinVar database (https://www.ncbi.nlm.nih.gov/clinvar/, SUB11294080).
